# Supplementary material for: Global marine phytoplankton dynamics analysis with machine learning and reanalyzed remote sensing
Source: PeerJ. 2024 May 8;12:e17361. doi: 10.7717/peerj.17361 (PMC11088370; doi:10.7717/peerj.17361)
Supplement: Supplemental Information 1 [file peerj-12-17361-s001.pdf]

Table S1. The Copernicus dataset statistical summary with respective symbols and their full forms

Item	Full-Form	Min	Max	Mean	Standard Deviation	Unit
spco2	Surface CO_2	27.6343	47.6918	37.09361	3.492898	Pa
o2	Dissolved Oxygen	194.507	244.464	206.7071	8.010823	$mmolm^{-3}$
no3	Nitrate	0.002294	16.1816	0.193351	0.678482	$mmolm^{-3}$
po4	Phosphate	2.92E-05	0.109444	0.002674	0.005743	$mmolm^{-3}$
phyc	Total Phytoplankton	0.799526	4.84236	1.328597	0.320024	$mmolm^{-3}$
si	Dissolved Silicate	1.44312	15.2757	3.825409	0.951438	$mmolm^{-3}$
ph	pH	7.96545	8.1759	8.065236	0.036145	-
fe	Dissolved Iron	0.000148	0.005593	0.001102	0.000499	$mmolm^{-3}$
mlotst	Density Ocean Mixed Layer Thickness	10.5289	129.093	27.94306	17.53611	m
thetao	Temperature	16.6903	31.0023	26.58887	2.554278	$°C$
bottomT	Sea Floor Potential Temperature	3.83077	29.2195	7.946663	7.265639	$°C$
vo	Northward Velocity	-1.24699	1.55522	0.052364	0.264279	ms^{-1}
uo	Eastward Velocity	-1.1298	1.27384	-0.00881	0.230234	ms^{-1}
so	Salinity	29.0002	36.9503	36.12722	0.506528	$1e^{-3}$
zos	Sea Surface Height	-0.47762	0.786462	-0.03472	0.178039	m
